# Validation of ‘Variable Number of Tandem Repeat’-Based Approach for Examination of ‘*Candidatus* Liberibacter asiaticus’ Diversity and Its Applications for the Analysis of the Pathogen Populations in the Areas of Recent Introduction

**DOI:** 10.1371/journal.pone.0078994

**Published:** 2013-11-05

**Authors:** Luis A. Matos, Mark E. Hilf, Jianchi Chen, Svetlana Y. Folimonova

**Affiliations:** 1 University of Florida, Department of Plant Pathology, Gainesville, Florida, United States of America; 2 Instituto Dominicano de Investigaciones Agropecuarias y Forestales, Santo Domingo, Dominican Republic; 3 U.S. Department of Agriculture - Agricultural Research and Services, Fort Pierce, Florida, United States of America; 4 San Joaquin Valley Agricultural Sciences Center, U.S. Department of Agriculture - Agricultural Research and Services, Parlier, California, United States of America; University of Padova, Medical School, Italy

## Abstract

Citrus greening (Huanglongbing, HLB) is one of the most destructive diseases of citrus worldwide. In South Asia HLB has been known for more than a century, while in Americas the disease was found relatively recently. HLB is associated with three species of ‘*Candidatus* Liberibacter’ among which ‘*Ca.* Liberibacter asiaticus’ (*C*Las) has most wide distribution. Recently, a number of studies identified different regions in the *C*Las genome with variable number of tandem repeats (VNTRs) that could be used for examination of *C*Las diversity. One of the objectives of the work presented here was to further validate the VNTR analysis-based approach by assessing the stability of these repeats upon multiplication of the pathogen in a host over an extended period of time and upon its passaging from a host to a host using *C*Las populations from Florida. Our results showed that the numbers of tandem repeats in the four *loci* tested display very distinguishable “signature profiles” for the two Florida-type *C*Las haplotype groups. Remarkably, the profiles do not change upon passage of the pathogen in citrus and psyllid hosts as well as after its presence within a host over a period of five years, suggesting that VNTR analysis-based approach represents a valid methodology for examination of the pathogen populations in various geographical regions. Interestingly, an extended analysis of *C*Las populations in different locations throughout Florida and in several countries in the Caribbean and Central America regions and in Mexico where the pathogen has been introduced recently demonstrated the dispersion of the same haplotypes of *C*Las. On the other hand, these CLas populations appeared to differ significantly from those obtained from locations where the disease has been present for a much longer time.

## Introduction

Citrus greening or Huanglongbing (HLB) is considered to be one of the most economically important citrus diseases around the world. HLB is the cause of losses of millions of citrus trees in many citrus growing areas wherever the disease has been reported [1], [2]. The disease was first described in the 18^th^ century in India [3], [4]. Later it was found in southern China in the 19^th^ century where the disease was referred to as “yellow shoot disease” [2], [5]. Since then, HLB has been reported in Taiwan, Japan, Indonesia, and others countries in Asia, and in South Africa [2], [6], [7], [8], [9], [10]. In Americas, the first report of HLB was in 2004 in Sao Paulo, Brazil [11]. In 2005 the HLB disease was found in Florida, USA [12]. After the initial finds, the disease has spread all over the Caribbean and Central America countries in a relatively short period of time. In 2007 it was found in Cuba [13], followed by detection in the Dominican Republic in 2008 [14], Belize and Puerto Rico in 2010 [15], [16] and almost simultaneously in Costa Rica, Nicaragua, Honduras, and Guatemala in Central America [17], Mexico in North America [18], and Jamaica [19].

HLB is associated with three species of ‘*Candidatus* Liberibacter’: ‘*C*. Liberibacter asiaticus’ (*C*Las), ‘*C*. Liberibacter americanus’ (*C*Lam), and ‘*C*. Liberibacter africanus’ (*C*Laf), which belong to the alpha subdivision of Proteobacteria [20]. Among these three species, *C*Las is widely distributed around the world, while *C*Lam appeared to be restricted to Brazil, yet it was recently reported in Hunan, China [5], and *C*Laf is present in Africa and Saudi Arabia [2], [10]. The bacteria could be transmitted by graft-propagation of infected material and by psyllid vectors, which are responsible for spreading the infection in the field: Asian citrus psyllid *Diaphorina citri* that transmits both *C*Las and *C*Lam and African psyllid *Trioza eryitreae* that is responsible for *C*laf transmission under natural conditions [2], [21].

*C*Las is thought to cause HLB in Florida. In the infected plants the bacterium invades most of the plant parts: leaves, stems, flowers, fruits, seed coats, and roots where it resides within phloem sieve elements [22], [23], [24], [25], [26]. The infected trees develop groups of leaves that show asymmetric blotchy-mottling or other chlorotic patterns and produce small, lopsided, poor tasting fruit [1], [2], [3], [27], [28]. Typically, trees die within a few years after the appearance of the initial symptoms. Thus far, no citrus species have shown resistance to HLB. Different citrus species, however, have different levels of tolerance to the disease, with sweet orange (*C. sinensis* (L.) Osbeck) and grapefruit (*C. paradisi* Macf.) being most sensitive to the disease [28], [29].

The detection and identification of *C*Las has been conducted using the sequence of 16S/23S rRNA and the outer membrane protein (*omp*) genes. In addition to these genes, the *rplKAJL-rpoBC* operon sequence, *nusG-rplK*, and bacteriophage-type DNA polymerase region [30], [31], [32], [33] have been used for genetic differentiation of *C*Las. The completion of the *C*Las genome sequence [34] has allowed identifying different regions of the genome with variable number of tandem repeats (VNTRs) [35], [36], [37], [38], [39]. Such VNTRs also known as microsatellites or short sequence repeats have been found in the genomes of various eukaryotic and prokaryotic organisms and represent tandem repetitive DNA sequences with a motif length of 2 to 6 or more base pairs (bp) [40]. VNTRs are among the most variable types of DNA sequences in the genome and are derived mainly from variability in length rather than nucleotide differences in the primary sequence [41]. After the development of polymerase chain reaction (PCR) methodology in the 1980's, the analysis and genotyping of VNTRs' polymorphism became a preferred approach in genome mapping and in population genetics studies [41], [42]. Although functions of most VNTRs are still unknown, some of them such as those of humans have been associated with regulatory functions and were shown to be markers of predisposition to a number of inheritable diseases [40], [43]. With prokaryotes, most studies were done using medically relevant microorganisms, and those demonstrated the involvement of VNTRs in bacterial adaptation as well as in pathogenesis [reviewed in 40,42].

One of the first studies on characterization of the variation in *C*Las populations using VNTR-based approach was done by Chen et al. in 2010 [39]. Analysis of 174 samples from Guangdong, China and Florida, USA using tandem repeats of AGACACA in the CLIBASIA _01645 locus in the *C*Las genome showed that populations of the bacterium in these two geographical regions are significantly different. Additionally, a possibility of the presence of two distinct populations of *C*Las in Florida was suggested. These data, however, were obtained based on the analysis of a single locus, which may not provide enough information for discrimination between different strains of *C*Las. More recently, a number of studies conducted by several research groups were published that identified additional *loci* containing hyper-variable sequence regions in the *C*Las genome that could be used for differentiating strains of the bacterium [35], [36], [37], [38]. Although these publications demonstrated the potential of VNTR-based approach for examination of *C*Las variability, an important question that needed to be addressed in order to validate this approach was: how stable are these repeats upon multiplication of the pathogen in a host over an extended period of time and upon its passaging from a host to a host? Obtaining an answer to this question was one of the objectives of this work. The presence of two different populations of *C*Las in Florida, which was confirmed in this study, provided an opportunity to examine the stability of VNTRs in the four *loci* of the *C*Las genome after passage of the pathogen in citrus and psyllid hosts. In continuation, we conducted an extended analysis of *C*Las populations in different locations throughout Florida and in several countries of recent introduction of the HLB pathogen, including the Caribbean, Central America regions, and Mexico and compared those with isolates from locations where the disease has been present for a much longer time such as China and Japan.

## Materials and Methods

### Collection of samples for examination of CLas populations in different regions

Examination of the composition of *C*Las populations in this work was conducted based on the analysis of 390 samples. Among those, 81 sample represented DNA extracts produced from plant tissue harvested from individual HLB-infected trees maintained in the greenhouse at the Citrus Research and Education Center, University of Florida, Polk county, Florida, USA (7 samples) or from trees grown in citrus orchards in Polk, Hendry, Marion, and Alachua Florida counties (29, 5, 20, and 20 samples, respectively). The greenhouse-grown trees used in this study were graft-inoculated with HLB-infected tissue as described in the reference 28, and the establishment of infection in the trees was confirmed by PCR analysis with *C*Las-specific primers as explained below. Collection of samples from field trees was conducted on the farms belonging to the University of Florida in Polk, Marion, and Alachua Florida counties that have been established for research purposes. Thus, collection of those samples did not require a special permission. In Hendry County samples were collected from a grove that is owned by United States Sugar Corporation/Southern Gardens Citrus with prior permission of the owner. Field studies did not involve endangered or protected species. Harvested tissue consisted of midribs from at least five leaves with typical HLB-related symptoms coming from individual plants. The tissue was processed for nucleic acid extraction followed by PCR analysis to confirm infection of the corresponding trees as described below. The rest of the samples analyzed represented DNA extracts of tissue collected from individual infected field trees of mostly sweet orange or grapefruit varieties grown in different locations throughout Florida (97 samples), eight countries in Central America and the Caribbean region (170 samples), Mexico, Brazil, China, and Japan (28, 2, 9, and 3 samples, respectively) kindly provided by other researchers (see in the Acknowledgements).

### DNA Extraction

Leaf midribs collected from leaves showing typical HLB-related symptoms were used for DNA extraction according to the procedure described earlier [44], [45]. Briefly, 250 mg was pulverized using a Kleco tissue pulverizer (Kinetic Laboratory Equipment Company, Visalia, CA 93292) in 2.5 ml extraction buffer (100 mM Tris-HCL pH 8.0; 50 mM EDTA; 500 mM NaCl; 10 mM dithiothreitol). 1300 µl was transferred to a 1.5 mL Eppendorf tube, 90 µl 20% SDS was added, and the mixture was incubated at 65°C for 30 minutes, followed by addition of 500 µl of 5 M potassium acetate, incubation on ice for 20 min, and subsequent centrifugation for 10 min at 15 rpm. Five hundred µl of the supernatant was precipitated with 500 µl of isopropanol. The DNA pellet was washed with 500 µl of 70% ethanol, dissolved in 100 µl of RNase-free water, and kept at −20°C for further analysis.

Psyllid DNA was extracted from individual psyllids using DNeasy Blood & Tissue Kit from Qiagen (Venlo, Netherlands) according to the manufacturer's procedure.

### Primers, PCR, and cloning

As a first step, all DNA extracts were subjected to PCR analysis using HLBaspr primer set [46] to confirm infection of the corresponding trees from which tissue was collected with the *C*Las bacterium. All reactions were done in triplicate with positive, healthy, and water controls. This was followed by examination of the number of repeats in *loci* within the *C*Las genome containing AGACACA, TACAGAA, CAGT, and TTTG motifs that were described previously [36], [38], [39]. Note that these motifs were designated as 005, 001, 002, and 077, respectively, in the reference 36. Herein, however, in order to improve the readability of the manuscript, in particular, the readability of the data presented in the tables, we refer to the same motifs as Motifs A, B, C, and D, respectively. The examination was conducted via PCR analysis using primer sets listed in Table 1.

**Table 1:** Primers for characterization of tandem repeats in four *loci* of the genomes of ‘*Candidatus* Liberibacter asiaticus’ isolates.

Motif	Sequence	Genome position	Primers' sequences 5′- 3′	Reference
A	AGACACA	354493–354527	(+) gacatttcaacggtatcgac	[39]
			(−) gcgacataatctcactcctt	
			(+) ttgaaggacgaaaccgatgg	this paper
			(−) cctgtacgaggtttgatcag	
B	TACAGAA	255591–255646	(+) gaagtagctctgcaatatctga	[36]
			(−) ggtgaattaggatggaaatgc	
			(+) cgcctacaggaatttcgttacg	[38]
			(−) tctcatcttgttgcttcgtttatcc	
C	CAGT	537729–537760	(+) ttgataatatagaaagaggcgaagc	[36]
			(−) tccatacccaaaagaaaagca	
D	TTTG	655277–655332	(+) gactgatggcaaaagatgg	[36]
			(−) agacacgccaaacaaggaat	

PCR reactions were carried out using the SpeedSTAR HS DNA polymerase from TaKaRa (Clontech Laboratories, Inc. Madison, WI). Each reaction mixture of 25 µl contained 2.5 µl of 10× Fast Buffer I, 2 µl of 2.5 mM dNTPs, 0.5 µM of each primer, 0.125 µl of *Taq* DNA polymerase (5 U/µl), 16.38 µl of water, and 0.2 µg DNA template. The following PCR conditions were used: 4 min at 94°C, followed by 30 repetitive cycles with 30 s at 94°C, 45 s at 55°C, and 30 s at 72°C, and a final step of 5 min at 72°C. PCR products were analyzed in a 2% agarose gel stained with ethidium bromide. For sequencing analysis PCR products were excised from a gel and extracted using a Geneclean Kit III from MP Biomedicals (Ohio, USA) according to the manufacturer instructions. DNA fragments were then cloned into pGEM-T vector (Promega Corp., Madison, WI). In general, for each plant sample 5 to 15 clones generated for each locus were subjected to sequencing analysis.

### Evaluation of the stability of VNTRs after passage of the *C*Las bacterium through citrus and psyllid hosts

A greenhouse-grown Duncan grapefruit (DG) plant infected with *C*Las haplotype HA (see details on *C*Las haplotypes characterization in the Results) was used as a source of inoculum to transmit the pathogen to other citrus plants as well as a source for psyllid acquisition of the pathogen. Tissue from the plant was used to graft-inoculate young seedlings of sweet orange Madam Vinous (MV) and DG. Additionally, the source DG plant was introduced in a cage with healthy psyllids (young adults) where those were allowed to feed for at least a week. Some of the psyllids were collected and used for DNA extraction followed by PCR analysis for the presence of *C*Las as discussed above. Two of the *C*Las-positive psyllids were subjected to further analysis for the number of VNTRs in the four *loci* containing Motifs A, B, C, and D as described above. The remaining psyllids that fed on the infected DG source plant were placed into another cage with young healthy seedlings of six different citrus species (DG, MV, *C. macrophylla* Wester, Sun Chu Sha and Clementine mandarins, sour orange) and allowed to feed on these receptor plants for two weeks. After this period of time the plants were sprayed with an insecticide (Malathion 50% E.C./Parafine Horticultural Oil from Southern Agricultural Insecticides, Inc., Palmetto, Florida, USA) to kill the psyllids and moved to the greenhouse where they were maintained and observed for the development of symptoms during 10–18 months along with graft-inoculated plants. Plants that developed an infection as was confirmed by PCR with *C*Las-specific primers were further used for analysis of VNTRs in the same four *loci*. In additional parallel experiments we used two field trees, DG and mandarin that were shown to be infected with the HB *C*Las haplotype, as inoculum source for subsequent grafting of two DG and two MV receptor plants, which then were allowed to develop infection over 10–18 months and used for further analysis. In all these experiments, for each of the four *loci* eight to ten clones obtained from DNA extracted from each receptor plant or each psyllid were used for sequencing in order to determine the number of repeats for each motif.

In order to evaluate the stability of those repeats after a period of five years we used plants and DNA extracts that remained from an early experiment conducted in 2007 in greenhouse facilities at the CREC [28]. Two plants that were used in that experiment - *C. macrophylla* (Cmac1) and *C. micrantha* Wester (Cmi) - remained in the greenhouse in 2012. The DNA extract produced in 2007 from the Cmac1 plant was stored at −20°C. No DNA prepared in 2007 from the Cmi plant was available. For this reason, we selected an additional DNA extract produced from another Cmac2 plant in 2007. Importantly, all three plants, Cmac1, Cmac 2, and Cmi, were graft-inoculated at the same time in 2007 using the same HLB inoculum source [28]. To assess whether VNTR profiles changed over a period of 5 years, DNA was extracted from the remaining Cmac1 and Cmi plants and subjected to further analysis for the number of repeats in the four *loci*. The four *loci* profiles were then compared using DNA prepared from Cmac1 and Cmac2 in 2007 and DNA extracted in 2012 from Cmac1 and Cmi plants.

DNA extracts from non-inoculated MV and DG seedlings were used as negative controls to demonstrate lack of amplification with primers used to amplify *C*Las DNA. As another control, the primers used to amplify the four *loci* within *C*Las genome were tested against *C*Lam DNA to show their specificity to *C*Las regions only. Bacterial population differences were analyzed by χ^2^ test.

## Results and Discussion

### Characterization of Florida *C*Las populations using four *loci* containing VNTRs

Previous analysis of the locus containing AGACACA tandem repeat in samples obtained from HLB-infected field citrus trees in Florida conducted by Chen et al. [39] suggested that two different *C*Las populations could have been introduced in the state. In our work we extended examination of Florida *C*Las populations using three additional *loci* with VNTR polymorphisms. Initially, 18 samples that included three samples from HLB-infected trees propagated in the greenhouse and 15 samples obtained from trees growing in the field in different locations in Polk county were evaluated based on the analysis of four *loci* containing Motifs A, B, C or D (Table 1). Seven samples were found to contain Motif A 4, 5 or 6 times, with most of the samples having the motif repeated 5 times (Table 2). In 11 of the 18 samples the same motif was found 13 times in the majority of the samples, while two samples had the motif repeated 12 or 14 times. Analysis of the Motif B showed that the first seven samples described above contained Motif B repeated 9 or 10 times, and in the remaining eleven samples the same motif was present 15 or 16 times. Motif C was found repeated 8 times in the first set of seven samples and 9 times in the other set of the eleven samples. Finally, the repeat D was found 14 times in the seven samples and 8 times in the rest of the samples (Table 2).

**Table 2:** Examination of tandem repeats in four *loci* of the genomes of ‘*Candidatus* Liberibacter asiaticus’ isolates from Florida.

Sample	No. of Repeats				*C*Las
	Motif A	Motif B	Motif C	Motif D	Haplotype
1	5	9	8	14	HA
2	5	10	8	14	HA
3	4	9	8	14	HA
4	5	9	8	14	HA
5	5	9	8	14	HA
6	5	10	8	14	HA
7	6	9	8	14	HA
8	13	16	9	8	HB
9	13	16	9	8	HB
10	13	16	9	8	HB
11	13	16	9	8	HB
12	13	16	9	8	HB
13	12	16	9	8	HB
14	13	15	9	8	HB
15	13	16	9	8	HB
16	14	16	9	8	HB
17	13	15	9	8	HB
18	13	16	9	8	HB

Therefore, the analysis of the four *loci* in those samples from Florida clearly demonstrated an existence of two distinct haplotypes or strains of *C*Las that showed different sequence profiles in the four regions in the genomes: one in which Motif A was repeated 4–6 times, Motif B – 9 or 10 times, Motif C – 8 times, and Motif D – 14 times and the other in which the same motifs were found to be present 12–14 times (Motif A), 15 or 16 times (Motif B), 9 times (Motif C), and 8 times (Motif D). Hereafter we refer to the first haplotype group as HA and to the second haplotype group as HB.

As a next step, we investigated whether there are differences in symptom expression between *C*Las isolates belonging to the two haplotypes using a limited host range of citrus varieties. No differences in symptoms were found upon examination of sweet orange and grapefruit field trees that were infected with either of the two *C*Las haplotypes. Leaves from field trees had typical blotchy mottle or zinc deficiency-like symptoms. Similar symptoms were also found in greenhouse trees in which the isolates were propagated, with no obvious differences noted between symptoms that developed upon infection with each of the two haplotypes of the bacterium.

### Validation of VNTR-based approach for differentiation of *C*Las populations

The data presented above along with the observations provided in earlier publications suggest that the analysis of VNTR-containing *loci* can be applied to examine diversity of the HLB pathogen populations. Our next goal was to further validate the usefulness of this approach and assess how stable are these repeats upon multiplication of the pathogen in a host over time or upon its passage from a host to a host. In order to evaluate the stability of tandem repeats, we assessed whether the number of repeats changes upon sequential passaging of the pathogen into new plant and psyllid hosts. The sequence composition for each of the four *loci* in *C*Las genome was analyzed using samples collected from the citrus plants that served as a source of inoculum for further propagation of the pathogen and compared with that in the receptor plants that were graft-inoculated using tissue obtained from the source plants. Two citrus varieties, DG and mandarin, were used as donor or inoculum sources in this experiment along with plants of several additional varieties that were used as receptor plants (Tables 3, 4, 5). The sequence composition in the four *loci* was analyzed in samples from psyllids that became infected after feeding on the source plants as well as in receptor plants that were inoculated with viruliferous psyllids. In all cases, when isolates of the HA or HB haplotypes were used as inoculum sources, a number of repeats characteristic for a corresponding haplotype group was found in all four *loci* in samples from the grafted receptors plants or from psyllids after the pathogen acquisition. In other words, for either *C*Las haplotype there were no changes in its VNTR “signature profile” after the pathogen was transmitted to a new plant or acquired by a psyllid vector (P>0.1; Tables 3, 4, 5). A typical “plus one/minus one” variation in the number of repeats of Motifs A and B was seen in samples from both source and receptor plants, while no variation in the number of tandem repeats of Motifs C and D was noted within a particular haplotype. For the HA group, Motifs A and B were present in most samples 5 and 9 times, respectively. Some proportion of samples contained Motif A repeated 4 or 6 times (28%) and Motif B 10 times (26%; Table 3). Most samples of the HB type isolates showed presence of these motifs 13 or 16 times, respectively, with a minor proportion of samples having Motif A repeated 12, 15 or 17 times and Motif B 14 or 15 times (Tables 4–5; more data on a repetition frequency for a particular motif is presented in Table S1).

**Table 3:** Analysis of the stability of tandem repeats upon transmission of the pathogen from Duncan grapefruit infected with HA haplotype of ‘*Candidatus* Liberibacter asiaticus’.

Sample	No. of Repeats			
	Motif A	Motif B	Motif C	Motif D
DG-GH^a^	5 (10/10^e^)	10 (9/10)	8 (5/5)	14 (9/9)
		9/(1/10)		
Psy-1^b^	5 (3/6)	9 (6/6)	8 (4/4)	14 (5/5)
	4 (3/6)			
Psy-2^b^	5 (2/4)	9 (8/8)	8 (6/6)	14 (4/4)
	6 (2/4)			
DG^c^	5 (4/6)	9 (6/7)	8 (5/5)	14 (5/5)
	6 (2/6)	10 (1/7)		
MV^c^	5 (4/5)	9 (7/7)	8 (5/5)	14 (5/5)
	6 (1/5)			
Receptor plants^d^	5 (5/8)	N/A	N/A	N/A
	6 (3/8)			

a *C*Las-infected greenhouse-grown Duncan grapefruit (DG-GH) used as inoculum source for graft or psyllid transmission of the pathogen.

b Psyllids that acquired the pathogen after feeding on DG-GH plant.

c DG and MV plants graft-inoculated using tissue from the source DG-GH.

d Receptor plants (DG, MV, *Citrus macrophylla*, Sun Chu Sha and Clementine mandarins, sour orange) that became infected upon psyllid transmission of the bacterium from the source DG.

e Number of clones contained a particular number of repeats out of total clones sequenced.

**Table 4:** Analysis of the stability of tandem repeats upon transmission of the pathogen from field Duncan grapefruit infected with HB haplotype of ‘*Candidatus* Liberibacter asiaticus’.

Sample	No. of Repeats			
	Motif A	Motif B	Motif C	Motif D
DG-Field^a^	13 (9/9^c^)	16 (9/10)	9 (9/9)	8 (9/9)
		15 (1/10)		
DG^b^	13 (10/10)	16 (8/9)	9 (9/9)	8 (9/9)
		17 (1/9)		
MV^b^	13 (19/20)	16 (13/13)	9 (10/10)	8 (13/13)
	14 (1/20)			

a *C*Las-infected field-grown Duncan grapefruit (DG-Field) used as inoculum source for graft transmission of the pathogen.

b DG and MV plants graft-inoculated using tissue from the source DG-Field.

c Number of clones contained a particular number of repeats out of total clones sequenced.

**Table 5:** Analysis of the stability of tandem repeats upon transmission of the pathogen from field-grown mandarin infected with HB haplotype of ‘*Candidatus* Liberibacter asiaticus’.

Sample	No. of Repeats			
	Motif A	Motif B	Motif C	Motif D
Man-Field^a^	13 (7/8^c^)	16 (8/8)	9 (8/8)	8 (10/10)
	12 (1/8)			
MV^b^	13 (5/7)	16 (5/6)	9 (5/5)	8 (4/4)
	14 (1/7)	15 (1/6)		
	15 (1/7)			
CM^b^	13 (3/7)	16 (4/4)	9 (3/3)	8 (3/3)
	14 (4/7)			
DG^b^	13 (6/6)	16 (4/4)	9 (2/2)	8 (3/3)

a *C*Las-infected field-grown mandarin (Man-Field) used as inoculum source for graft transmission of the pathogen.

b MV, *Citrus macrophylla* (CM), and DG plants graft-inoculated using tissue from the source Man-Field.

c Number of clones contained a particular number of repeats out of total clones sequenced.

The experiments above allowed us to assess whether there is a certain correlation between a particular *C*Las haplotype and the ability to infect citrus hosts. Evaluation of samples obtained from three citrus varieties, DG, MV, and *C. macrophylla*, demonstrated that both haplotypes could inhabit these varieties. Among 5 DG plants used, HA-type isolates were detected in two and HB - in 3 plants. Two MV sweet orange plants contained HA haplotype and 3 – HB. Three *C. macrophylla* plants were infected with HA and one with the HB haplotype.

In addition to evaluation of the stability of tandem repeats after passage through citrus and psyllid hosts, we were also interested in assessing whether a number of repeats could drastically change while the pathogen is present in a host during an extended period of time. In the experiments described above, sampling and analysis of the receptor plants inoculated using inoculum from source trees containing a particular *C*Las haplotype was conducted at 18 months after inoculation. The fact that the pathogen found in the receptor plants had the same VNTR profile as that in a plant used for inoculum suggested that these VNTRs are stable at least as long as the time frame tested (Tables 3, 4, 5). In addition, a study in which the repeats were evaluated over a period of time of 5 years was conducted. This study was done using a limited number of greenhouse-propagated HLB-infected plants remained from an earlier experiment that was carried out in 2007 [27] and DNA extracts obtained from these plants in 2007 and stored at −20°C as well as new extracts produced from some of the plants that were still present in the greenhouse. As a result, no changes were found in the profiles of the tandem repeats upon the analysis of the four genomic *loci* of the pathogen among DNA samples obtained at the time points, which had 5 years interval (P>0.1; Table 6).

**Table 6:** Analysis of the stability of tandem repeats over a period of five years.

		No. of Repeats			
Sample	DNA extraction date	Motif A	Motif B	Motif C	Motif D
Cmac1	05/22/07	5 (6/6)	9 (3/4)	8 (4/4)	14 (3/3)
			10 (1/4)		
Cmac2	10/24/07	5 (6/8)	9 (4/4)	8 (4/4)	14 (3/3)
		4 (2/8)			
Cmi	05/15/12	5 (5/5)	9 (2/2)	8 (2/2)	N/A^a^
Cmac1	03/15/12	5 (8/8)	9 (6/9)	8 (10/10)	14 (6/10)
			10 (3/9)		

a N/A, not assayed.

### Distribution of *C*Las haplotypes in Florida Counties

**Figure 1:**
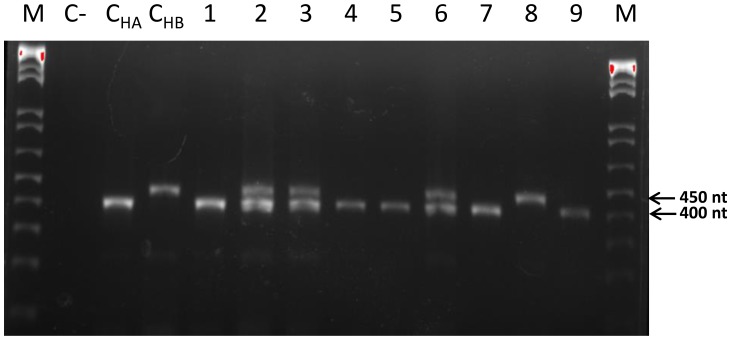
Polymerase chain reaction analyses of ‘*Candidatus* Liberibacter asiaticus’ isolates from Florida using primer set that amplifies Motif A-containing locus. Letters and numbers on top designate the following lanes: M, DNA markers; C-, control DNA from a healthy plant; C_HA_ and C_HB_, positive controls obtained by using DNA from plants known to be infected with the HA or HB haplotypes, respectively; 1–9, various samples from Florida. Note double bands in lines 2, 3, and 6. Numbers on the right indicate DNA fragments sizes.

The distribution of the two *C*Las haplotypes among eleven Florida counties was examined using 178 *C*Las-positive samples that were subjected to a VNTR-based analysis using two *loci* containing AGACACA (Motif A) and TACAGAA (Motif B) tandem repeats. The primer set that amplifies a Motif A-containing region generates a 400 bp long-fragment when the motif is repeated 5 times and about 450 bp long-product when the repeat is present 13 times (Fig. 1). Similarly, for Motif B, another primer set used in this work amplifies a 300 bp-fragment when the repeat appears 9 times and a product of around 350 bp in size when this repeat is present 16 times. This allows differentiate between the two haplotypes by analyzing the amplified PCR products in the agarose gel. Among the samples tested, 89 samples produced only 400 bp-long and 300 bp-long products in PCR reactions with the primers specific for Motifs A and B, respectively, indicating that the corresponding trees were infected with the HA haplotype of *C*Las (Table 7). Sixty four samples generated 450 bp and 350 bp-long fragments with the respective primers suggesting the presence of the HB haplotype (Table 7). Interestingly, in-gel analysis of PCR products amplified using DNA extracts from the remaining 25 samples and primers for either of the two motifs revealed presence of double bands corresponding to fragments specific to both HA and HB haplotypes (Fig. 1 and Table 7). This suggested that the trees from which the samples were taken could be infected with both haplotypes.

**Table 7:** Distribution of ‘*Candidatus* Liberibacter asiaticus’ haplotypes in Florida counties.

			Haplotype		
County	Location	No. of Samples	HA	HB	HA+HB
Polk	C^a^	40	7	24	9
Indian River	E	15	13	1	1
Charlotte	SW	12	10	0	2
Highlands	SC	15	12	3	0
Lake	C	15	9	1	5
Marion	NC	20	4	16	0
Hardee	SC	15	10	1	4
Hendry	S	5	5	0	0
St. Lucie	E	6	4	0	2
De Soto	SW	15	12	1	2
Alachua	N	20	3	17	0
Total		178	89	64	25

a Geographical location of counties in Florida: C, Central; N, North; S, South; W, West; E, East.

The two haplotypes of *C*Las appeared to have different distribution throughout Florida. According to our data, the HA haplotype is widely distributed in all sampled counties, except Polk, Marion, and Alachua counties where HB was more prevalent (Table 7).

The initial detection and, possibly, introduction of the HLB pathogen in Florida occurred in Miami-Dade County [12]. An earlier study by Chen et al. [39] showed wide distribution of the *C*Las haplotype that contains 5 repetitions of the Motif A in this region, the haplotype, which we refer here to as HA. In our work, the plants from an old experiment described in the above section that discusses the VNTRs' stability (Table 6), which were inoculated with tissue collected in the field near the site where the disease was first found [28] were shown to contain the HA haplotype. As shown in our work, HA is prevalent in the counties located in the south part of Florida (Table 7). Interestingly, the second haplotype HB is currently the most dominant *C*Las type in Polk County situated in Central Florida and in the two counties, Marion and Alachua, located north of the former county. These observations support a hypothesis proposed earlier on multiple introductions of the HLB pathogen [38], [39] and suggest that the introduction of second *C*Las haplotype could have occurred somewhere in the central region of the state, and from there it continued to spread to other areas. The presence of the HB haplotype, indeed, was determined in single or mixed infections in all counties sampled except in Hendry County, indicating that the spread of this haplotype is increasing with the time.

### Characterization of HLB populations in the Caribbean and Central America Countries

HLB was reported in most of the Caribbean and Central America countries a few years after it was found in Florida. To examine whether pathogen populations in these countries are similar to those found in Florida, we analyzed samples from eight countries in the Caribbean and Central America regions and Mexico. Two *loci* that contain Motifs A and B were used to evaluate 198 samples collected in those countries.

**Figure 2:**
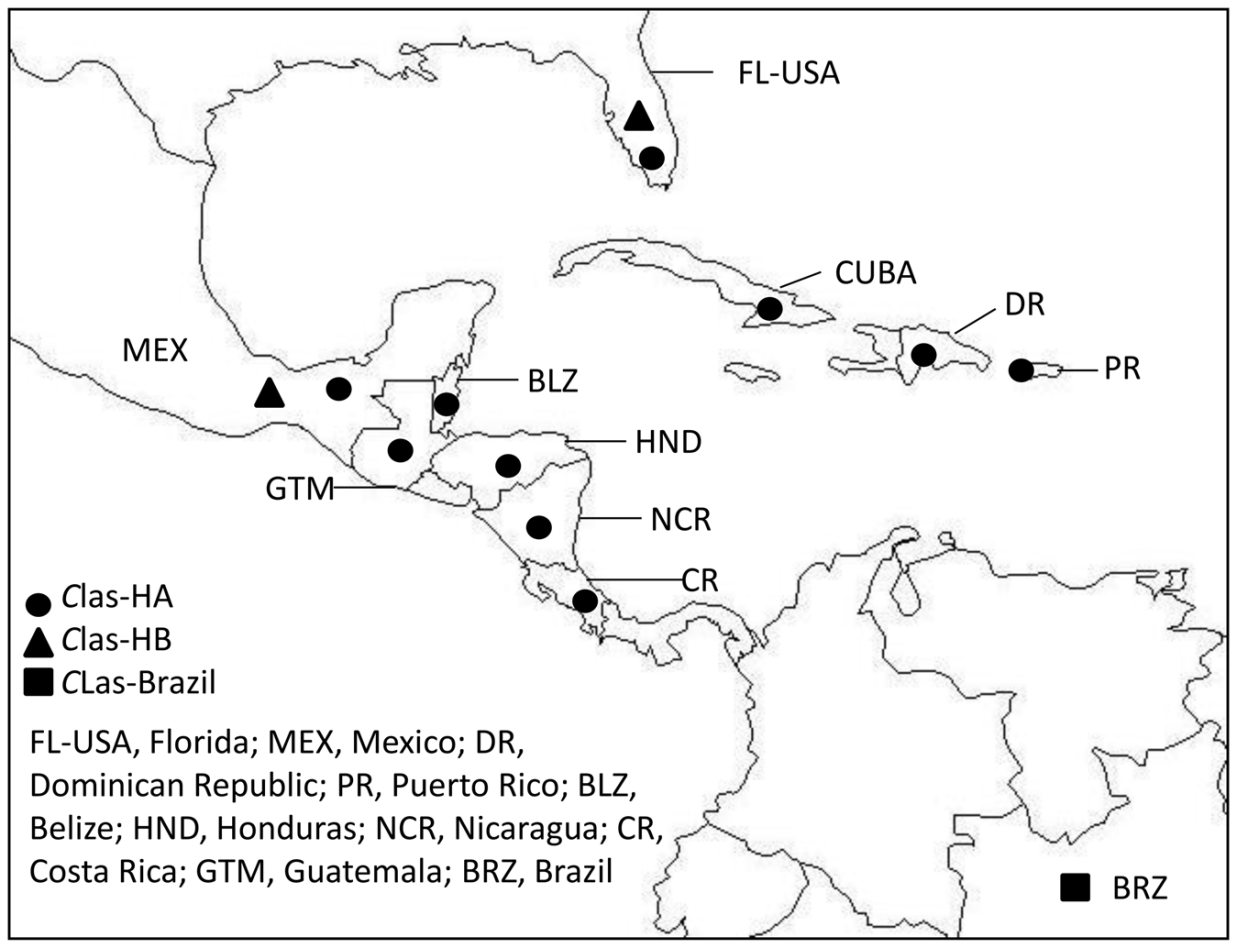
Distribution of ‘*Candidatus* Liberibacter asiaticus’ haplotypes in Florida, USA, Brazil, Mexico, and eight countries in the Caribbean and Central America region.

All samples from the countries tested, with the exception of samples from Mexico, produced only 400 bp-long and 300 bp-long products in PCR reactions with the primers specific for Motifs A and B, respectively, suggesting the uniform presence of the HA haplotype of *C*Las in these regions (Table 8; Fig. 2). Similar to Florida, both HA and HB haplotypes were found in Mexico. Large proportion of samples obtained from this region (11/28) amplified fragments characteristic to the HB haplotype group in addition to the samples (17/28) that showed VNTRs' profiles characteristic to the HA group (Table 8; Fig. 2). Further cloning and sequencing of the obtained PCR products confirmed those findings.

**Table 8:** Analysis of ‘*Candidatus* Liberibacter asiaticus’ populations in the Caribbean and Central America countries and Mexico.

Country	No. of Samples Tested	No. of Samples with Haplotype HA^a^	No. of Samples with Haplotype HB
DR	64	64	0
Costa Rica	21	21	0
Nicaragua	21	21	0
Belize	20	20	0
Honduras	10	10	0
Puerto Rico	13	13	0
Guatemala	20	20	0
Mexico	28	17	11
Cuba	1	1	0
Total	198	187	11

a Analysis was done based on examination of the number of repeats in Motifs A and B.

### Comparison of the two Florida-type *C*Las Populations with those from other geographical areas

Compared to being a century-old disease in South Asia, HLB is relatively new to Americas where it was initially found in Brazil following by the detection in Florida, the Caribbean and Central America. To assess how HLB populations in the latter three regions relate to those present in other areas, we analyzed several additional samples obtained from Brazil, China, and Japan and compared with the samples discussed above. According to our results, there is some similarity between Brazilian isolates and the HB-type isolates found in Florida and Mexico. Most common numbers of repetitions found in Motifs A, B, C, and D for the HB *C*Las haplotype are 13, 16, 9, and 8, respectively (Table S1). For the Brazilian isolates used here, those were 15, 18, 9, and 8 for the same motifs, respectively (Table 9). The isolates coming from Japan and China tested appear to be very different from isolates in Florida and the Caribbean and Central America countries (Tables 8 and 9). However, because the comparison was done based on a limited number of samples from the first two regions, we cannot rule out a possibility of the existence of additional haplotypes that could be more similar to those in Florida. To further address this possibility we compared VNTR profiles of the isolates studied here with those analyzed in the publication by Katoh et al. [36]. This analysis seemed to be particularly relevant due to the fact that in our work we used the same four *loci* as those initially described in the latter study. None of the 80 Japanese *C*Las isolates appeared to have tandem repeats profiles similar to that of the HA haplotype. On the other hand, some similarity could be seen between the HB haplotype and a number of isolates from Okinawa Main Island in which the Motifs A, B, C, and D were repeated 11–13, 12–15, 6–7, and 8–10 times, respectively, suggesting that the Florida HB-type isolates and those present in Japan could possibly share a common origin. The variations in the repeats number could then be explained by some changes occurred within the bacterium in order to adapt to different environments. However, further research is needed in order to understand what determines the variability of these regions in *C*Las genome.

**Table 9:** Examination of a few ‘*Candidatus* Liberibacter asiaticus’ isolates from China, Japan, and Brazil.

			No. of Repeats			
Country	Locality	Sample	Motif A	Motif B	Motif C	Motif D
China	Guangdong Sihui	1	6	25/26	6	7
		2	6	26	6	11
		3	7	20/21	6	11
	Guang Xi Yang Shou	1	6	14	7	7
		2	6	26	7	10
		3	6	24/26	7	10
	Guangxi Gongcheng	1	6	26	8	8
		2	8	26/27	7	12
		3	9	20/21	7	13
Japan	Japan	1	12	26	7	9
		2	11	11	7	7
		3	8	14	8	8
Brazil	Sao Paulo	1	15	18	9	8
		2	15	18	9	8

In summary, our results suggest that the numbers of tandem repeats in the four *loci* tested display very distinguishable “signature profiles” for the two Florida-type *C*Las haplotype groups. For each of these groups some minor (plus one/minus one repeat) variation could be found in the number of tandem repeats located in the two of the four *loci* tested (Motifs A and B), while the other two tandem repeats (Motifs C and D) remain invariable. Remarkably, the VNTR-based “signature profiles” do not change upon passage of the isolates in citrus and psyllid hosts as well as upon multiplication of the pathogen within a host over a period of time. This suggests that VNTR-based approach represents a valid methodology for differentiating between different types or haplotype groups (could be also referred to as strains) of *C*Las and provides a valid approach of analyzing the pathogen populations in various geographical regions.

The two *C*Las haplotype groups have different distribution in Florida citrus farms. Interestingly, these haplotypes appear to differ significantly from those obtained from the areas in South Asia and tested in this study. However, in those regions the disease has been present for more than a century, and the pathogen could have evolved over time into multiple distinct lineages increasing its diversity. Therefore, there is likelihood that types of *C*Las, which are more similar to Florida-type isolates, exist in those countries and even could have been a source of the pathogen that was introduced into the USA. Interestingly, the pathogen haplotypes distributed in Florida were found in countries of the Caribbean and Central America region and in Mexico. Whether those arrived into these areas at a time around the introduction of the pathogen into Florida yet were not detected until recently or have been brought at a different time remains to be understood.

## Supporting Information

Table S1 Polymorphism of VNTRs in four *loci* of two ‘*Candidatus* Liberibacter asiaticus’ haplotypes. Table represents a summary of the results obtained from examination of the number of Motifs A, B, C, and D repeats in the samples tested in this work.
(DOCX)
