# Supplementary material for: Validation of ‘Variable Number of Tandem Repeat’-Based Approach for Examination of ‘Candidatus Liberibacter asiaticus’ Diversity and Its Applications for the Analysis of the Pathogen Populations in the Areas of Recent Introduction
Source: PLoS One. 2013 Nov 5;8(11):e78994. doi: 10.1371/journal.pone.0078994 (PMC3818501; doi:10.1371/journal.pone.0078994)
Supplement: Table S1 — Polymorphism of VNTRs in four loci of two ‘Candidatus Liberibacter asiaticus’ haplotypes. Table represents a summary of the results obtained from examination of the number of Motifs A, B, C, and D repeats in the samples tested in this work. (DOCX) [file pone.0078994.s001.docx]

Table S1. Polymorphism of VNTRs in four *loci* of two ‘*Candidatus* Liberibacter asiaticus’ haplotypes^a^

| Motif | Haplotype | No. of clones^b^ | No. of Repeats |
| --- | --- | --- | --- |
| A | HA | 92/139 | 5 |
|  |  | 31/139 | 6 |
|  |  | 16/139 | 4 |
|  | HB | 69/86 | 13 |
|  |  | 11/86 | 14 |
|  |  | 5/86 | 12 |
|  |  | 1/86 | 15 |
| B | HA | 51/57 | 9 |
|  |  | 6/57 | 10 |
|  | HB | 50/54 | 16 |
|  |  | 4/54 | 15 |
| C | HA | 45/45 | 8 |
|  | HB | 46/46 | 9 |
| D | HA | 44/44 | 14 |
|  | HB | 52/52 | 8 |

^a^Table represents a summary of the results obtained from examination

of the number of Motifs A, B, C, and D repeats in the samples tested in this work.

^b^Number of clones contained a particular number of repeats out of total clones sequenced.
